# Orthopoxvirus Genome Evolution: The Role of Gene Loss

**DOI:** 10.3390/v2091933

**Published:** 2010-09-15

**Authors:** Robert Curtis Hendrickson, Chunlin Wang, Eneida L. Hatcher, Elliot J. Lefkowitz

**Affiliations:** 1 Department of Microbiology, University of Alabama at Birmingham, BBRB 276/11, 845 19th St S, Birmingham, AL 35222, USA; E-Mails: curtish@uab.edu (R.C.H.); eneidao@uab.edu (E.L.H.); 2 Stanford Genome Technology Center, Stanford University, 855 California Ave, Palo Alto, CA 94304, USA; E-Mail: wangcl@stanford.edu

**Keywords:** poxviruses, orthopoxviruses, variola virus, evolution, bioinformatics

## Abstract

Poxviruses are highly successful pathogens, known to infect a variety of hosts. The family *Poxviridae* includes Variola virus, the causative agent of smallpox, which has been eradicated as a public health threat but could potentially reemerge as a bioterrorist threat. The risk scenario includes other animal poxviruses and genetically engineered manipulations of poxviruses. Studies of orthologous gene sets have established the evolutionary relationships of members within the *Poxviridae* family. It is not clear, however, how variations between family members arose in the past, an important issue in understanding how these viruses may vary and possibly produce future threats. Using a newly developed poxvirus-specific tool, we predicted accurate gene sets for viruses with completely sequenced genomes in the genus *Orthopoxvirus*. Employing sensitive sequence comparison techniques together with comparison of syntenic gene maps, we established the relationships between all viral gene sets. These techniques allowed us to unambiguously identify the gene loss/gain events that have occurred over the course of orthopoxvirus evolution. It is clear that for all existing *Orthopoxvirus* species, no individual species has acquired protein-coding genes unique to that species. All existing species contain genes that are all present in members of the species *Cowpox virus* and that cowpox virus strains contain every gene present in any other orthopoxvirus strain. These results support a theory of reductive evolution in which the reduction in size of the core gene set of a putative ancestral virus played a critical role in speciation and confining any newly emerging virus species to a particular environmental (host or tissue) niche.

## 1. Introduction

Poxviruses are a family of viral pathogens known to infect a variety of organisms including insects, reptiles, birds and mammals. The wide distribution of poxviruses in nature suggests that an ancestral virus to this family might have been able to infect a common ancestor of vertebrates and invertebrates [1]. Based on some of these differences in host range, the *Poxviridae* family of viruses is subdivided into two subfamilies, the *Chordopoxvirinae* (ChPV), which infect vertebrates, and the *Entomopoxvirinae*, which infect insects. Each of these subfamilies is further subdivided into genera based on shared characteristics such as host range, morphology, antigenicity, and sequence similarity [2]. Host range represents one of the significant major phenotypic differences between members of the *Poxviridae* family [3], and there have been suggestions that one of the major evolutionary driving forces of this virus family has been co-speciation with their hosts [4,5]. Species in the *Avipoxvirus* genus only infect birds, though abortive infections can occur in other animals [6]. Members of the genus *Capripoxvirus* infect ruminants, including cattle, sheep, and goats [7]. *Suipoxvirus* species infects only swine [8] and *Leporipoxvirus* species infect only rabbit (leporid) species [9]. Viruses in the *Parapoxvirus* genus have a broader host range infecting animals in the superorder *Laurasiatheria* and may also occasionally infect humans [10,11]. Members of the *Yatapoxvirus* genus infect primate species [12,13]. Cervidpoxviruses infect species of deer [14], while the lone species in the genus *Molluscipoxvirus* is a human-specific pathogen and only causes serious problems for immunosuppressed individuals [15]. Viruses in the species *Squirrelpox virus* infect various squirrel species [16], and Crocodilepox virus was isolated from the Nile crocodile [17]. Viruses belonging to the various species that are members of the *Orthopoxvirus* genus have a broad host range and infect a wide variety of mammalian species including humans [18,19]. Viruses that are members of the *Cowpox virus* species primarily appear to infect rodent species as their natural hosts, but can also infect many other mammals including humans [20]. Members of the *Monkeypox virus* species also have a wide host range, infecting humans, non-human primates, and other large animals, as well as a large variety of rodents [21]. In fact, rodent species may represent the natural host of monkeypox viruses. The host range of ectromelia virus is more restricted, infecting mice and moles [22], while taterapox virus infects gerbils [23]. Camelpox virus is known to only infect camels [24] and variola virus, the causative agent of smallpox, is specifically restricted to humans [25]. The origins and natural host of viruses belonging to the species *Vaccinia virus*, that includes viruses used as vaccines to eradicate variola virus from the human population, remain unknown. But in laboratories, vaccinia viruses are able to infect a variety of species, and there have been occasional outbreaks of vaccinia among bovine populations in South America [26,27]. The *Orthopoxvirus* genus also includes two species whose viruses are native to North American mammalian hosts [18]. Complete genome sequences for strains of these species, *Raccoonpox virus* and *Volepox virus* as well as the related skunkpox virus are not yet publicly available, so these viruses could not be included in any genome-sequence-based analysis. They appear to form a distinct clade well separated on a phylogenetic tree from all other orthopoxvirus species. As complete genomic sequences for these viruses become available, it will be important to include them in analyses such as those reported in this manuscript.

The limited host range of some poxviruses in contrast to the broader host range of other poxviruses suggests the existence of gene-specific determinants that are responsible for the various host-range phenotypes [19]. In addition, the consequences of infection can vary from inapparent or very mild illness for some virus-host combinations, to significant disease and high mortality rates for others [23,28]. Studies to better define and understand the evolution of poxvirus species are in many cases attempting to discover the important genotypic differences that are responsible for the phenotypic differences in host range and disease.

A genome sequence is considered as the ultimate genetic map defining a species [29]. DNA sequence data provides the key information for determining the phylogenetic relationships among species, which in turn provides the framework for comparative approaches for biological investigation. The availability of genome sequence data and improvements in methods to analyze these data supports the process of comparative genomics, which allows us to discern both the common and contrasting features between different virus species (and virus strains) at the genome level. Comparative genomics promises a much more thorough and systematic approach to understanding the genetic diversity of species that leads to their different phenotypic properties, and determining the functions of newly identified genes in one species by studying their counterparts in other species [30]. These analyses then proceed to reveal the evolutionary history of, and the relationships between, species [31].

Since the early 1990s, efforts to completely sequence the genomes of multiple poxvirus species and strains have reached the point where currently over 120 complete genome sequences are publicly available. Complete genomic sequences are now available for representative isolates of all genera and most species of the subfamily *Chordopoxvirinae* as well as a few strains that belong to taxa in the subfamily *Entomopoxvirinae*. Poxvirus genomes contain a single linear molecule of dsDNA ranging in size from approximately 133,000–134,000 base pairs for members of the *Parapoxvirus* and *Yatapoxvirus* genera [32,33], to almost 360,000 base pairs for Canarypox virus, a member of the *Avipoxvirus* genus [34]. The two telomeres at the ends of the dsDNA genome form covalently closed hairpin structures at the termini [35]. Near the termini are sequences responsible for concatemer resolution of replication intermediates as well as a variable series of direct, tandem repeat sequences [36]. Finally, the ends of the viral genome contain inverted terminal repeats (ITR) that vary in size between species [37,38]. These ITRs can be large enough to contain the coding region for multiple genes, and genes contained within the ITRs are present as diploid copies. For orthopoxviruses, the size of the ITRs range from approximately 200 to 500 base pairs for variola viruses that contain no genes within their ITR, to almost 12,000 base pairs for several vaccinia virus strains containing six diploid genes within their ITR.

The coding potential of poxvirus genomes ranges from approximately 133 genes in parapoxviruses and yatapoxviruses to 328 genes in canarypox virus [39]. Overall, genome organization and syntenic gene locations are consistently maintained throughout *Chordopoxvirinae* species with the exception of a large genome inversion in avipoxvirus genomes [40]. Since poxviruses replicate in the cytoplasm of host cells, they must code for all proteins and enzymes required for their own transcription and replication instead of relying on the host proteins present in the nucleus [25,41]. These viral genes, encoded in the central portion of the poxvirus genome, generally code for functions involved in basic viral replicative processes: proteins involved in transcription, DNA replication, and virion assembly and release. In orthopoxviruses, this central core region of the genome comprises approximately 75% of the complete sequence and is the most conserved region of the genome. Genes present towards either end of the genome are much more variable between species in terms of both nucleic acid and amino acid sequence as well as whether or not the genes are present or totally absent from the genome. Proteins coded for by the genes in these variable regions are largely involved in host interactions including host range, immunomodulation, and pathogenicity [42,43].

Based on the analysis of poxvirus genomic sequences, a number of different studies have established the evolutionary relationship of members within the *Poxviridae* family [1,4,23,39,44–48]. Evolutionary studies can focus on different aspects of the overall variability that has occurred in poxvirus genomes over the course of their evolutionary history and the mechanisms by which this variation is generated. These mechanisms may include nucleotide sequence variation resulting in single base changes and small insertions and deletions; acquisition of new genes and genetic material through recombination resulting in horizontal gene transfer (HGT); and the loss of existing gene function through the fragmentation and loss of genetic material. The consequences of this variation ranges from neutral changes, to changes in protein function due to amino acid variation, to loss of gene function due to gene fragmentation, to acquisition of new function through HGT, and also potentially to changes in gene expression due to changes in the regulatory motifs such as promoter sequences that control gene expression. Therefore studies that attempt to reconstruct the evolutionary history of poxvirus species must, for example, include phylogenetic inferences based not only on the comparison of sequence information, but also gene content to illustrate some of the higher-level evolutionary processes that influence poxvirus variation [45,47]. Therefore to fully explore the potential changes in genotypic-phenotypic relationships that have occurred in the poxvirus lineage requires that an accurate, and consistently predicted gene set be utilized to ensure that the complete genomic complement of each virus species is compared and contrasted with that of every other virus species. Unfortunately, few previous studies have reassessed the available public annotation of the gene sets for each viral isolate under study. Instead, these studies relied on the annotations that were published along with the GenBank record of the genome sequence. These GenBank annotations are in many cases known to be inaccurate, and have not been updated since the original sequence record was released [49,50].

Our goal in this present work is to predict and utilize a much more accurate gene set for orthopoxviruses, and together with comparative genomic approaches, to identify the genetic diversity between poxvirus species, not only on a sequence comparison level, but also at higher levels that include analyses involving comparisons between sets of functional, as well as fragmented and missing genes. We describe the development and use of bioinformatics tools that allow us to re-predict the gene set coded for by representative isolates of each orthopoxvirus species. We then use these new gene sets to more fully explore the important genotypic and coding differences between species that may lead to better explanations for some of the phenotypic differences that result in differences in host range, immunomodulation, and ultimately pathogenicity and disease.

## 2. Results and Discussion

### 2.1. Poxvirus Phylogeny

**Figure 1.**
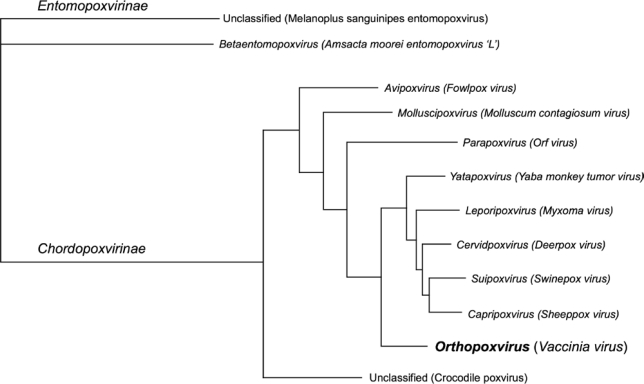
Gene sequence phylogeny of the family *Poxviridae*. Phylogenetic prediction based on an amino acid alignment of 20 conserved genes from representative virus isolates. Each terminal node is labeled with the genus name; and the type species for each genus is provided in parentheses. Unclassified viruses have not yet been assigned to a taxon. The *Orthopoxvirus* genus, analyzed in this manuscript, is highlighted.

Evolutionary analysis of the complete *Poxviridae* family is difficult due to the extensive divergence seen in gene and sequence content as well as gene synteny differences when comparing members of the *Entomopoxvirinae* and *Chordopoxvirinae* subfamilies. In fact, species in separate genera within the *Entomopoxvirinae* subfamily show almost as much divergence between themselves as they do with ChPVs [51,52]. Nevertheless, high-quality alignments can be generated using a subset of well-conserved genes shared between each subfamily, and these genes can be used to assess the evolutionary history for the whole *Poxviridae* family of viruses. Figure 1 shows a Bayesian tree based on a concatenated set of aligned amino acid sequences of the 20 conserved genes that could be unambiguously aligned (see Supplementary Table 1 for a list of these genes). The tree was inferred based on the amino acid alignment to minimize artifacts that arise from tree construction based on nucleic acid alignments of sequences with biased base compositions. (The family *Poxviridae* contains viruses with base compositions ranging from approximately 18% GC for entomopoxvirus strains to 64% GC for parapoxviruses and molluscum contagiosum virus.) This tree clearly delineates the two *Poxviridae* subfamilies, and shows the phylogenetic relationships that exist between the *Chordopoxvirinae* genera.

Using datasets containing larger numbers of shared orthologous genes, it is possible to further increase the resolving power of these evolutionary analyses and support more detailed analysis of the evolutionary history of these viruses [53]. While 20 gene families could be unambiguously aligned to support the analysis provided above, at least 49 genes show some level of significant sequence similarity across the whole family. Within the chordopoxviruses at least 90 gene families exhibit significant homology across the subfamily, while all orthopoxviruses share a core set of approximately 174 genes, and some subset of the 214 genes present in cowpox viruses are shared between every strain of every species of virus belonging to the *Orthopoxvirus* genus (see below) [39].

To fully understand the biology of poxviruses—their replication cycle, host interactions, pathogenesis, epidemiology, and evolution—it is necessary to understand at the very least the functions of all proteins encoded by these viruses and their genotypic-phenotypic relationships. But to continue to explore gene function, an accurate set of genes must be available. And to compare and contrast the biology of multiple viruses, accurate gene sets must be available for each one of those viruses. Unfortunately, as one begins to explore the gene sets provided in the GenBank annotations of many of the poxvirus genomic sequence records, it quickly becomes apparent that many of these gene sets are inaccurate and contain many small gene fragments that are annotated as functional genes, while also missing other genes known to be transcribed, translated, and functional in these viruses. In addition, available information on the structure, function, and role of individual poxvirus genes and their contribution to the overall biology of the virus has been greatly enriched [25,54–57] since the first poxvirus genome sequence was completed in 1990 [58,59]. However there has been little re-annotation of previously annotated genomes either to correct inaccurate gene predictions or functional annotations, or to identify previously unrecognized genes. Therefore, inconsistencies and inaccuracies remain common in the published annotations for poxvirus genomes.

So to begin a more detailed analysis of the evolution of the orthopoxviruses at the gene level, we first needed to develop a consistent, objective set of bioinformatics analyses to inform the process of genome annotation. We developed a software tool that could be used in a semi-automated manner to run these analyses, and provide for the visualization of the results. This tool then supported the final step of human-directed, manual refinement of the genome annotation. This allowed us to consistently predict gene sets for representative strains of all orthopoxvirus species, and then utilizing these gene sets, compare and contrast the coding potential and evolutionary history of each of these viruses.

### 2.2. Poxvirus Genome Annotation

Computational prediction of gene structures in genomic sequence has been one of the most active areas of bioinformatics research and has resulted in the development and application of many novel and innovative algorithms [60,61]. Approaches to gene prediction can be roughly divided into three categories: statistical approaches to look for features that appear frequently in gene regulatory and coding regions and infrequently elsewhere; similarity-based approaches where sequence similarity to previously identified genes and protein products provides evidence that a particular open reading frame (ORF) by inference may code for a functional protein; and phylogenetic approaches that rely on the observation that conserved regions in aligned genomic sequences of diverse species are more likely to contain coding regions and other functional motifs. But while gene prediction algorithms have steadily improved over the years, the performance of any one individual algorithm is still far from satisfactory, even for predicting simple gene structures like those present in poxviruses where RNA splicing does not occur and all genes are coded for by single, intact open reading frames. Therefore our approach to poxvirus genome annotation involves the utilization of integrated strategies that combine many different algorithms and analyses to improve the overall performance of our gene prediction pipeline. To support this goal, we developed the poxvirus genome annotation system (PGAS) to facilitate the re-annotation of currently available sequences and the annotation of forthcoming sequence projects. Using this system, we have re-annotated representative virus strains of all species in the genus *Orthopoxvirus*.

#### The Poxvirus Genome Annotation System

**Figure 2.**
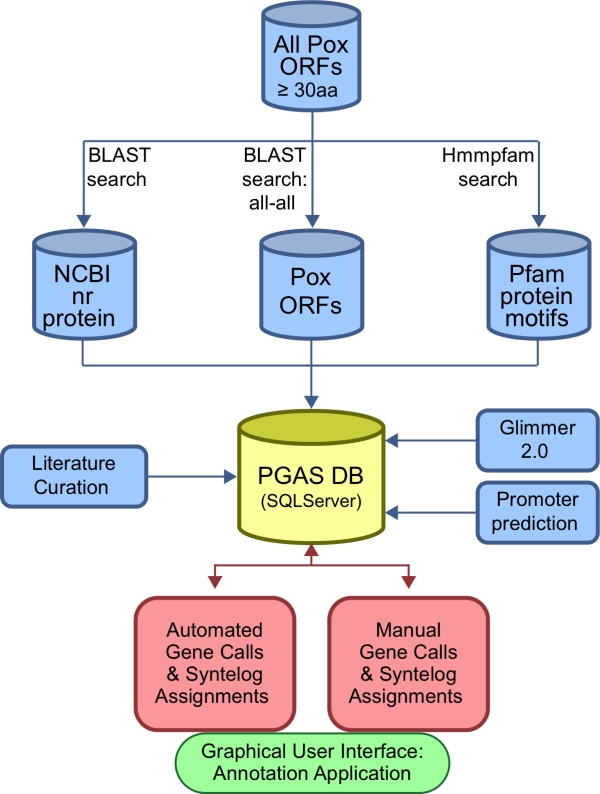
The Poxvirus Genome Annotation System (PGAS) design. The PGAS pipeline (blue) automatically runs the underlying analyses in parallel on a local high-performance computing cluster for each new genome. Results from those analyses are then loaded into the PGAS database (yellow). The process of making gene calls (red) is directed from a desktop java GUI application (green).

**Figure 3.**
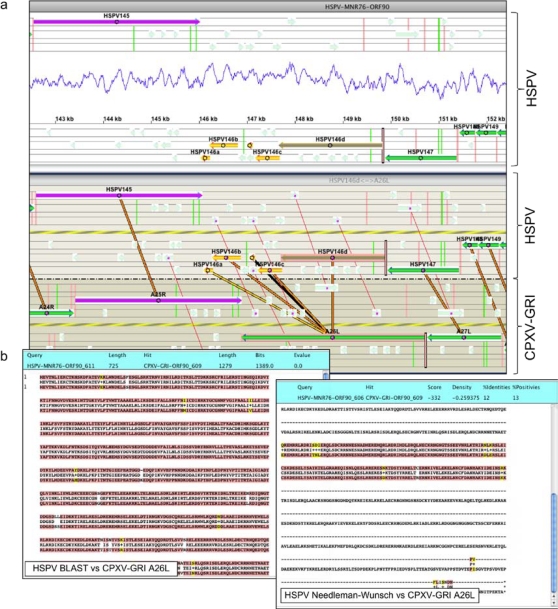

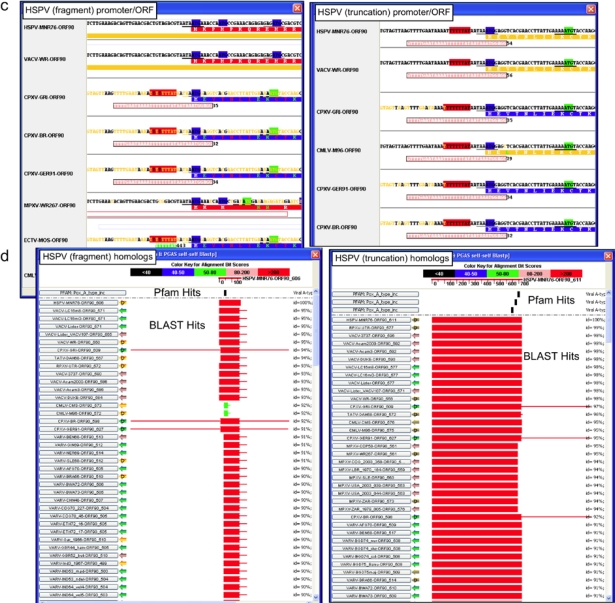
PGAS Screen Shots **(a)** Single genome display for HSPV and a dual-genome display of the same HSPV region and its homolog in CPXV-GRI. ORFs from all 6 translational reading frames are shown as horizontal lines, with putative, and as available, verified transcriptional promoters indicated by colored vertical lines. ORFs showing similarity by BLAST analysis are connected by red lines. ORFs that have been annotated into the same orthologous gene family are connected by thicker lines. **(b)** Results of the BLAST search between two ORFs (left panel). The results of a Needleman-Wunsch (global) alignment is displayed in the right panel. **(c)** Multi-genome homolog comparison of a representative ORF showing the predicted start of the coding region with potential ATG translation start sites highlighted. The 5′ untranslated region with predicted promoter sequences highlighted is also displayed. Early termination sequences from the upstream gene are indicated in red. Late promoters are colored pink. **(d)** Homologs across multiple genomes can also be displayed using a BLAST/Pfam viewer. Examples of fragmented (left panel) and truncated (right panel) genes are shown.

PGAS consists of a computational pipeline that runs each genome sequence through a series of analyses using a variety of computational algorithms that provide various types of information pertinent to gene prediction. The results of these analyses are available from a genome visualization tool that supports the annotation of individual genomes, and the comparative analysis of sequence features between genomes. The pipeline proceeds as follows: First, ORFs greater than or equal to 30 amino acids in all six frames are translated into peptides. Homology searches are carried out using BLAST [62] to search for similarities in the NCBI nr protein database or a database of poxvirus proteins. Similarities to known protein family motifs are detected using HMMPFAM to search for hits in the Pfam database [63]. To be functional, a poxvirus ORF must be transcribed, and transcription is regulated by specific promoter sequence motifs. Different promoter motifs exist that interact with virus-encoded transcription factors to support transcription at early, intermediate, or late times after infection. To predict early and late promoter motifs, a novel algorithm was developed based on interpolated Markov models [64–66]. This algorithm extended a simple weight matrix model of bases present at specific positions by providing for sequence dependencies between positions in an alignment of known promoter sequences. These models were then used to search for similar motifs present in the genomic sequence, and ranked those motifs according to the degree of similarity to the promoter model. Predicted promoters present upstream of an ORF then provide evidence that supports the expression of that ORF. Potential genes were also evaluated and scored according to their match to a poxvirus gene model constructed by the program Glimmer 2 [66]. All results were then loaded into a Microsoft SQL Server relational database to support manual inspection and curation of the results using a customized graphical user interface (GUI). Gene assignment decisions were based on the evaluation of multiple lines of evidence such as adjacency of a potential promoter sequence, a reasonable Glimmer score, a statistically significant hit to the NCBI nr sequence database or the Pfam database of amino acid motifs, and most importantly, conservation of the ORF between phylogenetically related species. In PGAS, all of these data provide evidence that can be easily visualized using the GUI. Figure 2 provides a flowchart of the automated pipeline that generates the PGAS data. Figure 3 shows an example of the PGAS GUI, displaying a comparison between homologous regions of the cowpox virus (CPXV-GRI) and horsepox virus (HSPV) genomes, where a gene intact in CPXV-GRI is fragmented in HSPV.

The screen shots provided in Figure 3 only show a few of the available screens. The PGAS GUI application contains multiple panels for displaying the various types of evidence that support the identification of a particular ORF as a coding gene. The gene layout panel (top panel of Figure 3a) displays the arrangement of ORFs in the genome and also displays a graph showing the base compositional bias (GC base frequency) along the genome. The dual genome comparison panel (lower panel of Figure 3a) shows similarities detected by a BLAST search between neighboring ORFs around orthologous genes in any two genomes. Using this panel, a user can visualize the similarity between any two selected genes by aligning them with the Needleman-Wunsch algorithm [67]. A separate panel displays predicted promoters, early transcriptional termination signals, and in-frame and out-of-frame ATG triplets. A similarity comparison panel shows hits to the Pfam and NCBI nr database for each potential gene. Finally, a gene up-date panel provides detailed information on each gene, including the sequence and its coding potential as evaluated by Glimmer 2.0. This panel is then used to annotate the gene, provide links to pertinent references in the literature, and make the final assignment as to its coding potential.

During the process of gene annotation, not only are gene assignments made and confirmed, but additional features of the annotations are also assessed. Most—if not all—previous poxvirus annotations assumed that the first codon in an ORF is the translation starting point. Although this happens to be correct for many genes, some genes may not follow this simple rule. By taking into account the position of predicted promoters, strength of the Kozak consensus sequence [68], and comparing orthologous genes in different species, PGAS can identify a more likely translation start site. Alterations to the translation start site for a gene is indicated in Supplementary Table 1. (Of course, the only definitive means of determining the actual translation start site is through N-terminal sequencing of the translated protein.) We also use the comparative analysis features of PGAS to assign all predicted genes to orthologous families. These are families of genes conserved across two or more poxvirus taxa that show significant sequence similarity and therefore allow for an inference of common function to be made. Since the genes present in these orthologous families also share a common genome location and gene order when comparisons are made between all orthopoxvirus genomes—shared gene synteny—we refer to these orthologous families as “syntelogs”: a contraction of “synteny” and “ortholog”.

**Figure 4.**
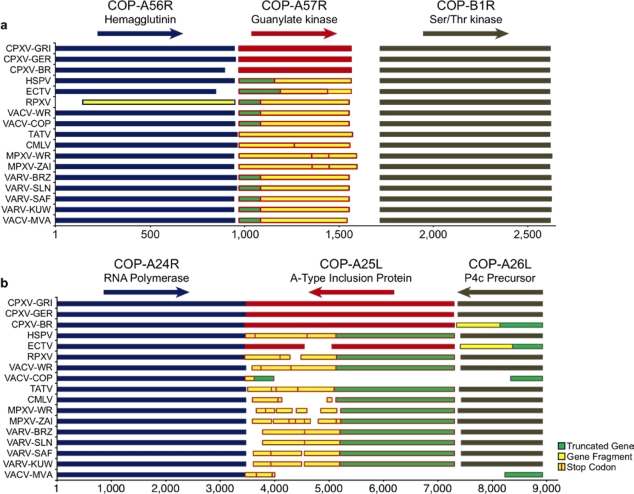
Fragmentation of orthopoxvirus genes **(a)** Fragmentation pattern of the guanylate kinase gene. **(b)** Fragmentation pattern of the A-type inclusion protein. Virus abbreviations are defined in Table 1.

While annotating poxvirus genomes, many small sequence fragments can be annotated as partial sequences or pieces of coding ORFs [69]. With PGAS, comparing orthologous genes and syntenic genome regions between two closely related strains or species can easily identify gene fragments. Figure 4 shows two examples of genes that are fragmented in some orthopoxvirus species but not others. In the first example (Figure 4a) a gene coding for guanylate kinase is present between a hemagglutinin gene and a gene coding for serine/threonine kinase. In cowpox viruses, the guanylate kinase protein is 197 amino acids in length, similar in size to the 198 amino acid guanylate kinase protein present in mice. In variola, vaccinia, and ectromelia viruses, there is a break in the ORF resulting in a truncated protein. In camelpox, monkeypox, and taterapox viruses, the gene is so fragmented, that no transcribed coding region has been annotated in PGAS. As seen in Figure 4b, cowpox viruses code for a 1,279 amino acid protein that contains multiple repeats of the A-type inclusion (ATI) protein motif. ATI proteins are believed to form protein aggregates in the cytoplasm of virus-infected cells and these inclusions may be involved in virion assembly [70]. ATI proteins are coded for by most ChPVs, but as seen in Figure 4b, the ATI gene is fragmented in many orthopoxviruses.

When annotating genes, it can be difficult to determine if any particular fragment should be annotated as coding for protein. To provide consistency, we developed defined criteria for gene annotation. In this study, if an ORF is intact at its 5′ end and retains a predicted promoter sequence, it is annotated as an intact gene if it is at least 80% or greater of the length of its intact counterpart. If an ORF is intact at the 5′ end and maintains a predicted promoter sequence, but that ORF would code for a protein that is less than 80% of the length of the intact orthopoxvirus protein, then it annotated as a truncated gene. Any ORF that has lost its predicted promoter and/or has been significantly truncated at its 5′ end is annotated as a fragmented gene. In most cases, we would not expect fragmented genes to be transcribed and/or translated into a functional protein product. If for any particular virus, no remnants of any significant sequence fragments can be detected for a particular gene, then that gene is annotated as missing in that virus. These criteria are admittedly somewhat subjective. And of course the only way to definitively assess the coding potential of any ORF is to experimentally determine if that ORF is transcribed and translated under a variety of *in vitro* and *in vivo* conditions.

### 2.3. Orthopoxvirus Gene Prediction

Using PGAS, we re-predicted and annotated the complete gene sets of representative strains of each orthopoxvirus species. The list of viruses that were analyzed is provided in Table 1, along with virus-specific information including the abbreviation used for that virus in the figures; the length of the viral genome; the length of the virus ITR regions; the total number of coding genes (intact plus truncated) predicted to be transcribed and translated into protein; and the number of genes present within the ITR and are therefore present as diploid copies in that particular virus. The GenBank accession number for the genome sequence along with the Pubmed ID of the publication describing the genome sequencing are also provided. The genome sequence analyzed for the Copenhagen strain of vaccinia virus is one base larger than that presented in its GenBank record (accession number M35027) due to an additional base inserted into the A2.5L gene. The absence of this base in the M35027 sequence is the result of a reported sequencing error [71,72].

The lengths of the ITRs reported in Table 1 should be considered to be approximate. These were compiled by determining the number of complementary bases that appear at each end of the reported genomic sequences. But for each genome, the beginning and end of the reported sequence starts and stops at different points near the termini of the linear genome. A few of the available sequences do contain the complete sequence up to (and through) the ends of the genome (e.g., VACV-WR), but most do not. So the true length of the ITRs is unknown, but in all cases the reported length is probably within a few tens, or at worst, a few hundreds of bases of the actual length.

**Figure 5.**
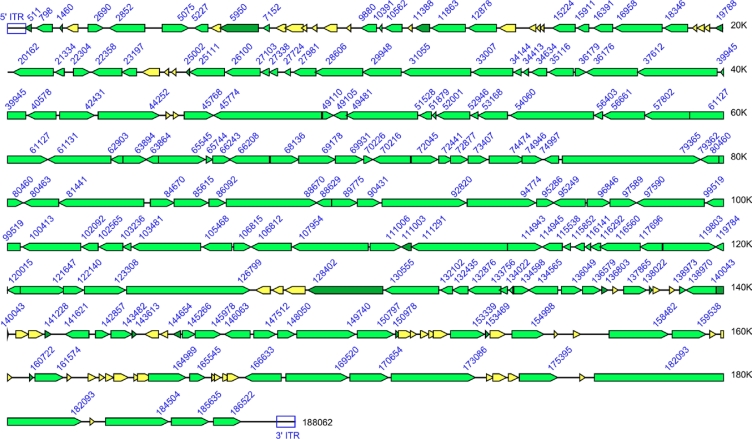
Variola virus strain Brazil 1966 Genome Map. Each arrow indicates the presence of an ORF within the VARV-BRZ genome. The arrow also designates the direction of transcription. Intact genes are colored using light green arrows; truncated genes by dark green arrows; and fragmented genes by yellow arrows. The numerical designations indicate the position of the last base of each ORF. The position of the ITRs at both ends of the genome is also indicated.

The complete set of genes annotated for each genome is provided in Supplementary Table 1 along with a functional identification for each gene. Figure 5 shows, as an example, the resulting gene map for Variola virus strain Brazil 1966. Intact genes are displayed in light green, truncated genes in dark green, and fragmented genes in yellow. As can be seen, most of the fragmented and truncated genes are present towards the ends of the genome, while most of the genes in the central region of the genome are intact. This pattern corresponds to that seen for all poxviruses with the conserved core set of genes that are responsible for basic replicative processes mostly intact; while the genes coding for proteins that interact with the host and that confer some of the unique biological properties of different viruses showing much more variability.

### 2.4. Comparative Analysis of Orthopoxvirus Gene Content

**Figure 6.**
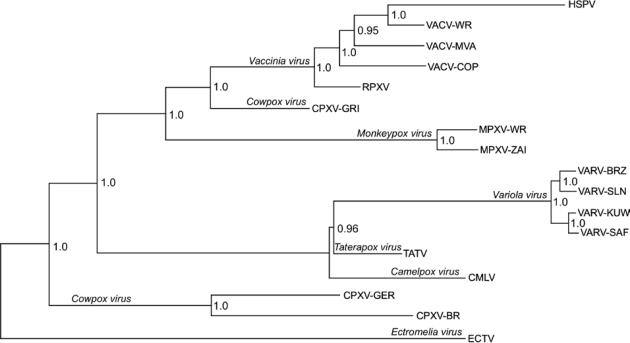
Gene sequence phylogeny of the genus *Orthopoxvirus*. Codon-aligned gene sequences of 141 genes from each indicated orthopoxvirus were used for phylogenetic prediction using Bayesian inference. Species names are indicated along the branch distinguishing each species clade, and strain names are provided at each terminal node. The numbers at each node provide the clade credibility values for each node—a measure of the confidence of the branching pattern for the indicated clade.

When compared at the sequence level, any two viruses from different species in the *Orthopoxvirus* genus share at least a 96% identity when compared at the nucleotide level over the length of the alignable region of their genome, while any two strains from the same species show at least a 99% nucleotide sequence identity [28]. This alignment includes the core region of the genome along with a portion of the more variable region up to the point at which differential copies of repeat sequences, large deletions, and the variable ITR regions prevent the construction of a reasonable alignment [24]. (The left end of the alignment extends from orthologous genes represented by VACV C7L, position 15,716 of the VACV-WR genome, to A51R at the right end of the genome at position 158,673). The presence of multiple, conserved genes in every viral genome provides us the opportunity to compare phylogenetic relationships based on a comparison of the aligned gene sequences of these viruses. As seen in Figure 6, strains of each virus species group together in separate clades. This tree is consistent with those reported in the literature and shows the fairly broad clade of strains that comprise the species *Vaccinia virus*, and the two separate clades present for both monkeypox viruses and for variola viruses. For monkeypox viruses, the lower pathogenicity Western African strains (represented by MPXV-WR) form a distinct clade from the higher pathogenicity Central African strains (represented by MPXV-ZAI). For variola viruses, as expected, the South American/Western African strains form a clade (VARV-BRZ, VARV-SLN) separate from the Asian/non-West African strains (VARV-KUW, VARV-SAF).

It is interesting to note that as has been previously observed, the three currently available completely sequenced strains assigned to the *Cowpox virus* species do not form one separate clade when analyzed based on their gene sequences. CPXV-GRI forms a branch that lies at a point near the base of the vaccinia virus lineage, while both CPXV-BR and CPXV-GER form a separate clade between the ectromelia virus lineage and the camelpox/taterapox/variola virus lineage. The assignments of these three strains of cowpox virus to a single species were based on shared non-sequence biological properties such as host of isolation, lesion, growth properties, and morphology. But based on sequence-only comparisons and existing demarcation criteria for *Orthopoxvirus* species, these viruses seem to clearly belong to two separate species. As discussed below, this demarcation into two species is not supported when comparing these viruses based on their gene content.

**Figure 7.**
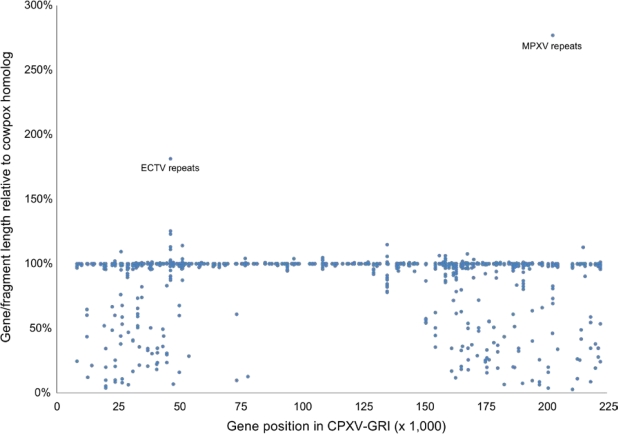
Comparison of orthopoxvirus gene lengths. The length of every annotated orthopoxvirus gene from the genomes listed in Table 1 was compared to the length of the corresponding cowpox virus ortholog. The length of each gene as a percentage of the length of the longest cowpox virus strain gene was plotted with respect to its genomic position in CPXV-GRI.

As emphasized above and in previous publications, when a direct comparison is made between shared orthologous gene families, sequence variation and gene variation is greatest towards both ends of the linear genome [39,44,46,47]. Examining differences in gene length for each orthologous family across the *Orthopoxvirus* genus further emphasizes this variation. Figure 7 plots the length of each orthologous gene as a percentage of the length of the corresponding cowpox virus gene that is used as a reference. A great deal of variability in gene (and protein) length is observed in genes that are coded for near the ends of these genomes, while much less variability is observed in the core central region of these genomes. (The few anomalous points that appear to be extremely long genes coded for by non-cowpox virus strains are due to genes in particular strains where small repeat sequences have been greatly expanded in number thus increasing the size of the overall gene.)

**Figure 8.**
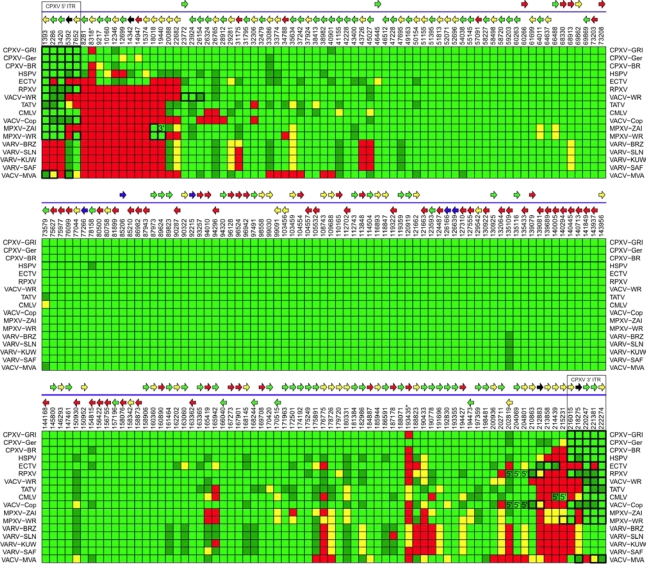
Comparative orthopoxvirus gene conservation map.

A graphical view of the genus-wide pattern of gene content is provided in Figure 8. Each column in the figure represents a unique orthopoxvirus gene family, which are ordered according to their syntenic position in CPXV-GRI. (The 214 columns representing unique syntelog gene families are split into three panels representing the left-third, middle-third, and right-third of the genomes.) Each row corresponds to a separate virus strain, and the strains are ordered according to the degree of conservation of orthologous family genes. Each cell within the figure is colored according to the annotation assigned to that gene for that virus within PGAS. Genes are colored according to their degree of conservation: intact (light green), truncated (dark green), fragmented (yellow), and missing genes (red). The map uses genes from CPXV-GRI as a reference to indicate genomic position of the end of each gene (vertical numbers above each column). The two genome positions marked with an asterisk * indicate that the corresponding ORF in CPXV-GRI is not present, and the numbers provided correspond to CPXV-GER gene stop location. Arrows above the gene family columns indicate the direction of transcription and are color coded according to experimentally determined and predicted expression temporality based on gene expression analysis of the corresponding gene in vaccinia virus [81]: early (green arrows), late (red), early/late (yellow), intermediate (blue), and unknown (black). Genes present in the ITR of any particular genome are outlined in a heavy black box. The five genes that are part of the cowpox virus ITR are repeated at either end of the figure to represent the fact that they are present in diploid copies in these cowpox virus strains. Genes labeled 3′ are part of a gene family that is present at the 5′ end of cowpox viruses, but is located near (but not a part of) the 3′ terminal ITR of the indicated genome. Genes labeled 5′ are part of a gene family that is present at the 3′ end of cowpox viruses, but is located near (but not a part of) the 5′ terminal ITR of the indicated genome.

**Figure 9.**
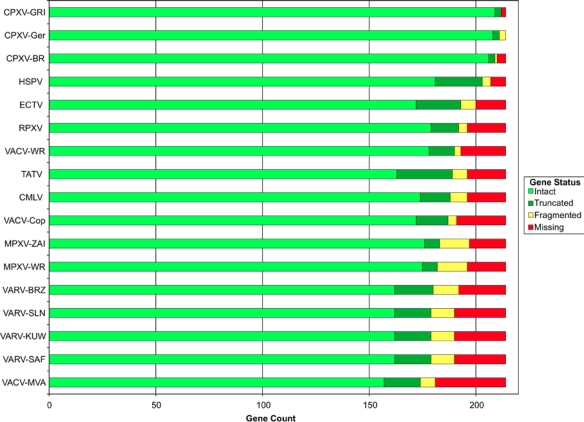
Gene loss summary. The number of intact (light green), truncated (dark green), fragmented (yellow), and missing (red) gene families is plotted for each virus strain.

The pattern observed of intact, truncated, fragmented, and missing genes in orthopoxvirus strains, further emphasizes the conservation of genetic material in the core region of poxvirus genomes, and the variability near the ends of the genome. Importantly, a pattern begins to become apparent in the degree to which genes are conserved for any particular strain and species. This is summarized in Figure 9 and Table 2 where a summary of the number of genes conserved, fragmented, and missing in each strain is provided. The three cowpox viruses contain every gene present in every other orthopoxvirus species. (This is another characteristic that these three strains have in common irrespective of the fact that their sequence-based phylogeny predicts that they belong to two separate species.) Therefore, given the reasonable inference that the progenitor to all current-day orthopoxvirus species contained all genes currently present in all of these species, then cowpox virus appears to be most like that progenitor virus, at least in terms of gene content. Viruses of every other orthopoxvirus species contain a subset of those genes.

While strains of cowpox virus contain an essentially complete set of gene family orthologs, variola viruses in contrast, contain the most restricted set of genes for any naturally-occurring orthopoxvirus. (Only VACV-MVA contains fewer genes, but MVA is an attenuated vaccine strain of vaccinia virus that was isolated following multiple passages *in vitro* [80].) Variola viruses contain a set of 162 intact and 17 truncated genes that would be predicted to code for functional protein. This is in contrast to cowpox viruses that code for up to 214 functional genes. It is interesting that viruses belonging to the orthopoxvirus species *Cowpox virus*, that infects the widest variety of host species, contains the largest number of genes of any species in the genus; while viruses belonging to the species *Variola virus*, code for the most restricted gene set, but are the most host restricted, and at the same time the most pathogenic of any other species in the genus.

**Figure 10.**
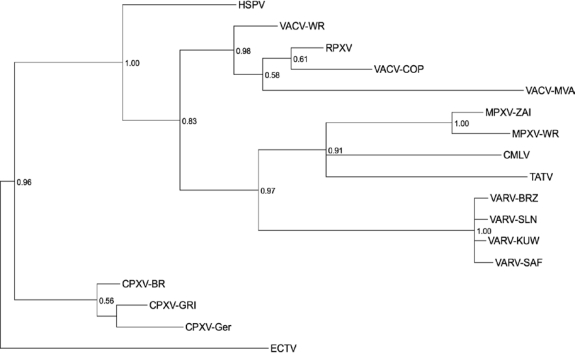
Gene content phylogeny of the genus *Orthopoxvirus*. A Bayesian phylogenetic tree inferred on the basis of similarities in gene content between virus strains. Strain names are provided at each terminal node. The numbers at each branch point provide the clade credibility values for each node—a measure of the confidence of the branching pattern for the indicated clade.

One additional method for assessing gene content differences between orthopoxvirus strains is to infer their phylogenetic relationship based on these gene content differences, and not based on the usual multiple sequence alignment [48,82–85]. Figure 10 shows a phylogenetic inference of virus strains based on their gene content. Each gene in each genome was coded as to whether or not it was intact, truncated, fragmented, or missing in that virus strain. Then this gene content matrix was used as input to the program MrBayes to calculate the gene content phylogeny. For pairwise genome comparisons based on this matrix, each orthologous syntenic gene was compared between each genome, and a mismatch was scored if the character states of the two genes being compared did not match. Then MrBayes was used to infer the most probably phylogenetic relationship based on these gene content comparisons.

While similar to the sequence-based phylogeny presented in Figure 6, there are important differences observed when comparing the two trees. In the gene content tree, the three cowpox virus strains form a common clade since each contains an almost complete set of intact orthopoxvirus genes. This is in contrast to the two clades seen when assessing their phylogenetic relationship based on sequence comparisons. Taterapox virus and camelpox virus still form a common clade, but now, they form a clade with monkeypox viruses instead of with variola viruses. Ectromelia virus continues to lie on the most extended branch of the tree, while most of the vaccinia virus strains analyzed form an extremely broad divergent clade similar to, but perhaps even more extended then that observed in the sequence-based tree. This is probably explained by the artificial nature of vaccinia viruses: while their natural origin remains unknown, current strains are essentially laboratory viruses passaged under a variety of *in vivo* and *in vitro* conditions. It is therefore not surprising that they would exhibit extensive variability not only in their sequence, but also in their gene content. It is interesting that based on sequence analysis, horsepox virus is clearly a strain of the species *Vaccinia virus* [78]. But based on gene content, horsepox virus forms a clade separate from the clade containing all of the other vaccinia virus strains. This is in contrast to the sequence-based tree where horsepox virus lies on an extended branch of the vaccinia virus clade (Figure 6). Horsepox virus was isolated from a natural outbreak of disease in horses, and is therefore the only vaccinia virus in this study that was obtained from a natural outbreak as opposed to being derived from artificial passage in the laboratory. Supporting this difference, the gene content of horsepox virus is much more similar to that of the cowpox virus strains than to the other vaccinia virus strains. These differences between the gene content of horsepox virus when compared to the other vaccinia virus strains, may reflect a history of infection in a “natural” host and the selection pressures of such natural passage *in vivo* may require a broader complement of host-interacting genes than for the “artificially”-passaged laboratory strains of vaccinia virus.

### 2.5. Discussion

To reliably evaluate the evolutionary history of any virus family, and to better understand the selection pressures that have influenced that evolution, it is necessary to begin with a reliable and consistently annotated set of genes predicted to be coded by viruses belonging to that family. The prediction of genes coded for by large DNA viruses is not possible solely by annotating ORFs larger than some arbitrary cutoff as an expressed gene. Therefore we have developed a poxvirus-specific gene prediction tool that is able, in a semi-automated manner, to streamline the gene prediction and annotation process for poxvirus genomes. PGAS is able to help refine the translation start point of a coding region; identify truncated genes; identify fragmented, possibly non-functional genes; and identify small, previously unrecognized genes. Using its semi-automated prediction pipe-line, the system can either predict the gene content of a newly sequenced poxvirus genome, or re-annotate a previously sequenced genome very efficiently, allowing information pertaining to the remaining ORFs to be inspected and assessed using the GUI inspection tool.

Over-prediction of genes has been a significant problem in previous poxvirus genome annotations. For instance, the initial annotation of the sequence of VACV strain Copenhagen uncovered 263 ‘potential’ genes, many of which are small and overlap with other genes [58,59]. By combining evidence from genome comparison, promoter prediction, similarity searches, and compositional analysis, we have found that a substantial number of previously annotated genes in many poxvirus species to be artifacts due to lack of information or to the limitations of previously employed gene prediction algorithms. Our current annotation of VACV-COP predicts that 187 genes may be expressed and functional in this virus strain. By applying integrated, consistent strategies as implemented in the PGAS tool, we were able to re-predict and annotate the gene sets for representative strains from all species with available complete genomic sequences in the *Orthopoxvirus* genus. Although the tool was developed for the prediction of poxvirus genes, it should be applicable to other viruses such as herpesviruses and prokaryotic organisms with a simple gene structure.

Inspection of poxvirus genomic sequences and their predicted gene sets emphasize three major mechanisms of variation that have occurred throughout their evolutionary history: (1) single base changes causing amino acid variation or variation in regulatory regions such as promoter sequences; (2) acquisition of new genetic information through horizontal gene transfer or gene duplication driven by the recombination of poxvirus genomic DNA with that derived from the virus host or other co-infecting pathogens; and (3) the gradual loss of genetic information and coding genes through progressive deletion of DNA sequence over many rounds of replication/infection. Single base changes may result in functional variation of existing proteins or changes in gene expression. HGT may result in the acquisition of new function. Gradual deletion will result in the loss of function. The key feature of all types of variation is that while they result from essentially random processes, to influence virus biology and evolution, they must provide some selective advantage that drives fixation within the virus genome. Our analysis of poxvirus genomes reveals the debris resulting from this continuing process of genome evolution. These truncated, fragmented, and missing genes that we observe, reflect the ongoing process of selection and fixation.

Features of poxvirus biology that might influence the ability of viruses to accommodate the loss of existing or acquisition of new genes are the structure of the virion and the organization of the genome. The virion structure must be flexible enough to contain a viral genome that can vary by at least 45,000 bases. The structure of the genome must be able to accommodate the insertion of significant lengths of new genetic material. The flexibility seen in poxviruses being able to introduce recombinant genes into poxvirus vectors emphasizes the ability of these viruses to accommodate new genetic information [86–88]. In addition, gene order in poxvirus genomes may not be as restricted by functional constraints as it is for other viruses, and can therefore be disrupted to a greater extent without deleterious effects [89]. A substantial number of genes unique to different poxvirus genera and species are found to be present near the ends of the virus genome. However, when confining the comparisons to the core regions of each genome, only a few unique genes are found in each representative species. Therefore, over the evolutionary history of these viruses, it appears that genes absolutely required for virus replication have “migrated” towards the central part of the virus genome, while genes with more peripheral functions, whose loss may be less disruptive to basic replicative processes, are located in the terminal regions of the viral genome. In this manner, evolution has provided for the segregation of poxvirus genes according to their role in virus biology. The central part of the genome represents the utility room of the virus without which nothing happens—the utility room houses all of the machines required to keep the virus functional, and in general, you do not mess with these machines. The terminal regions of the genome represent the parlor—the place where the guests first arrive. The parlor is where you hold the party where you entertain a wide variety of guests. If the guest proves to be useful, you invite them to stay awhile, and perhaps even give them a more permanent home. If the guest serves no useful purpose, you show them the door.

The absence of a viral fossil record makes it difficult to ascertain whether a taxa-specific gene is due to a recent gene acquisition event in that particular taxa, or is due to the loss of that gene from related taxa. But the presence of gene fragments can help infer which particular evolutionary process may have been responsible. Once the selection pressure to maintain gene function is lost, it appears that the sequence for the gene itself is also fairly rapidly lost through progressive deletion. Truncated genes, and especially gene fragments, are the result of that ongoing process.

The evolutionary process in orthopoxviruses exhibits two seemingly disparate features. On the one hand, species within the genus show extensive differences due to fragmentation and deletion of genes. On the other hand, the core genomic sequences of these viruses show very little variation, as reflected by the fact that more than a 96% nucleotide identity is seen throughout the core region of these genomes. But as demonstrated in the present study, major differences between viruses in the *Orthopoxvirus* genus predominantly involve the inactivation or loss of a number of genes originally present as a larger repertoire possessed by an ancestral virus similar to modern-day cowpox viruses. Therefore gene loss appears to be the predominant evolutionary process that drives the divergence of orthopoxvirus species. These changes in the non-core gene sets may lead to rapid changes in virus-host interactions resulting in divergence and speciation.

Variola virus, the etiologic agent of smallpox, contains the smallest genome and gene set of any other orthopoxvirus that circulates naturally in their host(s). This suggests that inactivation of genes may have reduced the ability of variola virus to propagate in alternative hosts, eventually restricting replication and transmission to just a single host: humans. It is interesting to note that cowpox viruses, with the largest orthopoxvirus genomes, have the widest host range of viruses belonging to the genus, while variola viruses, with the smallest genomes, have the most restricted host range. Cowpox viruses might be considered to be the sports utility vehicle of the poxvirus world—big lumbering beasts with many disparate functions, none of which is finely tuned, but all of which provide support for a wide range of uses. Variola viruses in contrast can be considered the Ferraris of the poxvirus world—small, streamlined, and tuned to do one thing well: infect and kill humans.

The last common poxvirus ancestor might have been able to infect early eukaryotic organisms, as reflected by their wide, present-day distribution and natural host range, which spans insects to mammals [1,4]. In spite of this wide distribution in the environment, many modern-day poxviruses have a tendency to exhibit a fairly narrow host range for any one particular species. During the evolution of the virus within a host, poxviruses may have lost the genes unnecessary for infection of that particular host species, keeping only those genes necessary to successfully parasitize that particular environmental niche, a process in part recapitulated by endosymbiotic bacteria [90]. These gene-loss events may have jeopardized the virus’ ability to infect other organisms, forming a natural host species barrier. Virus evolution due to gene loss may therefore represent one of the defining processes through which the basic biology of modern-day poxviruses is determined.

## 3. Experimental Section

### 3.1. Genome Sequences

For evaluation of the evolutionary history of the *Poxviridae* family, we chose a representative strain of the type species from each genus. For re-annotation and analysis of the *Orthopoxvirus* genus, we chose representative strains of each species in the genus where complete genome sequences have been determined (Table 1). Where distinct clades exist for any particular species (*Variola virus, Vaccinia virus, and Monkeypox virus*), we utilized members of each clade.

### 3.2. Multiple Sequence Alignments and Phylogenetic Tree Construction

For gene-based phylogenetic analysis, translations of the nucleotide sequences of the open reading frame of each gene were aligned using MEGA 4 [91] and the ClustalW algorithm [92]. Amino acid multiple sequence alignments (MSA) were used to generate corresponding codon-aligned nucleotide MSAs. The individual amino acid alignments or nucleic acid alignments were concatenated together into one large contiguous alignment for subsequent phylogenetic analysis. For the core orthopoxvirus genome nucleotide alignment, the alignment was generated using a combination of the programs MAVID and Multi-LAGAN [93,94]. The final computational alignment was then hand edited extensively to optimize the alignment. The alignment extends from base 15716 to base 158673 of the Vaccinia virus strain Western Reserve (VACV-WR) genome. This alignment starts with VACV-WR_021 and ends with VACV-WR_177 and corresponds to the VACV-Copenhagen (VACV-COP) genes C7L to A51R [79].

Phylogenetic inference of the family *Poxviridae* was based on an amino acid alignment of 20 conserved genes of virus isolates from representative species of each genus (Supplementary Table 1). For the sequence-based orthopoxvirus phylogeny, an alignment of codon-aligned nucleic acid sequences from 141 conserved genes was used (Supplementary Table 1). All trees were inferred by using Bayesian inference with Markov chain Monte Carlo methods as implemented by MrBayes [95]. The poxvirus amino acid tree was estimated by allowing model jumping between all fixed-rate amino acid models. For the nucleic acid sequence phylogeny, the most appropriate nucleotide substitution model was first determined using the program MODELTEST [96]. The best fitting model was a general time reversible (GTR) model of nucleotide substitution that allowed for gamma-distributed variation across sites with a proportion of invariable sites [95,97]. Tree analysis was performed for at least 100,000 generations with a sampling frequency of 100. Trees were constructed from the MrBayes run data after disregarding the initial 25% as burn in.

For the gene content phylogenetic inference, the MrBayes model utilized was the standard discrete morphological model with variable coding and 4 character states (0: intact, 1: truncated, 2: fragmented, 3: missing). Characters were unordered. The model assumed equal stationary state frequencies and equal substitution rates and the branch lengths were unconstrained, with all topologies equally probable. The prior was set to symmetric Dirichlet with fixed (–1.00) variance parameter. The cost to switch from any one state to any other was set to 1. Tree analysis was run for 200,000 generations with a sampling frequency of 100. Trees were constructed from the MrBayes run data after disregarding the initial 25% as burn in.

### 3.3. Poxvirus Genome Annotation System (PGAS)

Different gene prediction methods often examine different aspects of an actual gene, all of which may complement each other and yield better predictions. Therefore, in order to achieve optimum predictive capability, PGAS was designed to integrate as many useful gene prediction methods as possible as long as they individually provide some predictive capability and the algorithm that provides that capability is not also implemented in another method used in PGAS. PGAS contains four independent approaches: sequence similarity comparison, comparative genomic analysis, promoter detection, and a test of coding potential.

#### 3.3.1. Sequence Comparison

The genomes of living organisms have arisen through modifications of an array of ancestral sequences. Duplication with modification is a central paradigm of protein evolution, wherein new proteins and/or new biological functions are fashioned from earlier ones [98]. Similarity over an extended region of a sequence in most cases implies homology, or descent from a common ancestral gene [99]. The similarity of a region of the genome to a sequence that is already known to be transcribed is the single most powerful predictor of whether the newly annotated genomic sequence is part of a gene and is therefore transcribed into mRNA and translated into protein [100]. Gene prediction algorithms that take sequence similarity into account generally outperform those that do not [101].

However, two concerns have to be taken into consideration when using sequence comparison to predict gene content in a genome sequence. The first one is the quality of the sequence database. Since most sequences deposited into a database are based more or less on results from prediction and annotation by similarity, mistakes made early on will be propagated repeatedly from one sequence to the next. The second concern is the coverage of the sequence database and the sensitivity of the similarity-detecting algorithm. Biological sequence data may be both extremely redundant for some genes, but at the same time relatively sparse for others. Discovering closely related homologues is relatively straightforward due to the development of efficient similarity-search algorithms [62,102]. However, it is much harder to detect similarity between two distantly related sequences due to the accumulation of mutations. To address concerns about the quality of the sequence database and the inability to detect distant similarity by conventional pair-wise sequence comparison algorithms, PGAS uses similarity information as detected by BLAST [62] against common sequence databases such as the NCBI nr database, only as supporting evidence during manual inspection. Instead, PGAS relies heavily on detecting statistically significant matches between an ORF and the Pfam database of functional protein motifs. Pfam is a comprehensive collection of protein domain families curated by experienced biologists and bioinformaticists, with a range of well established uses including genome annotation [63]. In addition, each domain in the Pfam database represents an empirically derived estimate of all possible evolutionary changes for a protein of particular function, which then leads to identification of a much higher proportion of distantly related sequences [103] with a searching algorithm, HMMPFAM, based on a hidden Markov model (HMM).

The tradeoff of using HMM-based searches for increased sensitivity is the intrinsically slow nature of the Viterbi [104] or forward algorithm used in the search application. In addition, as the size of the publicly-available protein database continues to grow at a rapid rate, it also takes a significant amount of time to search these databases even with the relatively efficient BLAST algorithm. To solve this problem, PGAS uses our previously developed algorithm for deploying these searches on a Linux cluster [105].

#### 3.3.2. Genomic Comparative Analysis

Functional sequences are subjected to evolutionary selection. When two sequences are aligned, most of the observed differences are neutral, having no effect on the amino acid sequence of the encoded protein. Other mutations result only in a conservative change of one amino acid for another similar amino acid, or the changes may occur in regions of the protein that do not directly play a role in protein function and may therefore be more tolerant of change. In contrast, regions of a gene that are devoid of mutation may be conserved because the mutations may result in a loss of function and therefore be detrimental to the organism [106]. Using computer-based analysis to focus in on the genomic features that have been preserved in phylogenetically related species, researchers have been able to pinpoint the motifs responsible for function, such as protein coding regions and gene regulatory motifs.

Members of the subfamily *Chordopoxvirinae* share many basic features with each other. One significant feature is the overall conservation of gene synteny (genomic organization)—the pattern in which their genes are arrayed along the chromosome is almost identical when comparing most of the species within the ChPV subfamily (only the avipoxviruses show significant deviation due to a few large scale rearrangements within their genomes) [44]. On the other hand, in spite of this conservation, other aspects of genome composition have undergone dramatic changes. For example, the GC content in both the parapoxviruses [32] and molluscipoxviruses [107] is more than 60%, while it is only about 25% in Capripoxvirus species [7]. Synteny conservation at the gene level and disparity in GC content of genome sequences permit unambiguous identification of functional motifs including genes, because any similarity due to conservation of nonfunctional sequence is eliminated over time. Synteny conservation also allows the establishment of orthologous relationships among genes. The comparative genomic approach has also proven to be very powerful in detecting small genes that are conserved in several closely related species [108]. Unlike larger genes, the statistical signals to distinguish small functional ORFs from non-coding sequence are very weak and therefore these genes may simply be over-looked by more conventional approaches such as similarity searching and tests for codon-bias or biases in base composition.

PGAS detects similarity between any two ORFs in all sequenced poxvirus genomes with sensitive all-against-all BLAST searching. Two orthologous genes and those surrounding them in each species are displayed graphically in a panel in the PGAS tool, and are connected with lines indicating possible pairs of orthologs. By including similarity relationships for neighboring genes, the orthologous relationships can then be unambiguously resolved due to the conservation of genome organization. Since it is more difficult to detected similarities between two short genes using the BLAST algorithm, in PGAS a pairwise alignment between any two genes can be generated as desired using an included implementation of the rigorous Needleman-Wunsch algorithm [67].

#### 3.3.3. Promoter Prediction

The expression of poxvirus genes are controlled by a promoter sequence that lies upstream of the mRNA start site and initiating ATG codon. Just as one can reach the melon by following the vine, it is possible to use the predicted promoter sequences as a “signal”—by knowing the position of a promoter one knows at least the approximate starting point of the transcript, thus delineating one end of the gene. This information is also helpful in predicting a small gene, where weakness in the signal of the coding region prevents confirmation of whether it is expressed until experimental evidence is available. Although poxvirus promoters appear to be simple conserved sequence motifs, computational identification is far from a fait accompli. In general, promoter-prediction algorithms that are able to locate a reasonable percentage of true promoters, also give a high number of false-positive predictions [109]. To increase the reliability of gene and promoter prediction, PGAS combines these predictors to provide mutually supportive results. In addition, other types of information—such as adjacency to an ORF and reading frame compatibility of the first ATG codon following the transcription start site with as well as the inter-species conservation of both promoter and gene—are all taken in account.

The significant compositional features and inter-dependencies observed in vaccinia virus promoter sequences were incorporated into interpolated context models (ICMs) [65,66], which were then employed to predict promoters in the vaccinia virus genome, as well as all other orthopoxvirus genomes. By coordinating positional and compositional features and dependencies in a signal, ICMs lead to improvement in predictive capability compared to a simple weight matrix model (WMM). Temporal-specific promoter sequence models were built based on experimental data reported in the literature. For each ORF, the predicted promoter sequence, the coding region, and the poxvirus early gene transcriptional termination signal were projected onto a panel in PGAS, which also provides for visualization of the same information for orthologous genes in other species. Manual inspection was then carried out to verify the predicted promoter sequence and downstream open reading frame. For those ORFs with predicted promoters that show the predicted sequence located within the putative coding region, comparison between orthologous genes was used to resolve the conflict by either altering the translation start site to the next in-frame ATG triplet, or by reassessing the accuracy of the predicted promoter sequence.

#### 3.3.4. Characterization of Coding Potential Using Glimmer

The base composition of genes is strongly affected by evolutionary constraints, and therefore may be statistically “unfavorable” in the context of the whole genome. A DNA sequence that encodes protein is not a random chain of available codons for any particular amino acid, but rather an ordered list of specific codons that reflect the evolutionary origin of the gene as well as constraints associated with genome replication, gene expression and function [110]. A composition-based gene finding approach detects the statistical bias present in coding regions. In general, these tools are first trained with the coding regions that comprise a set of known, true genes for the species under study. Then this model is used to evaluate the coding potential of every ORF present in that species. A poxvirus gene consists of a single continuous open reading frame separated from the next gene by a short intergenic region—a pattern that is similar to a prokaryotic gene model. Glimmer 2.0, one of the available prokaryotic gene finders, is widely used for prokaryotic genome annotation [64]. In PGAS, Glimmer 2.0 was customized to evaluate the coding potential of each orthopoxvirus ORF using an isolate-specific model constructed from the ORFs in that isolate that have statistically significant hits to the Pfam database. The Glimmer score provides a useful clue in manually resolving ambiguous predictions.

#### 3.3.5. Semi-Automatic Gene Prediction

For efficiency, scalability, and consistency, automated processes are highly preferable to manual curation. However achieving both efficiency and accuracy simultaneously is not yet possible given the limitations of the current gene prediction algorithms. For that reason, PGAS is designed to operate in a hybrid mode in which the results from an initially automated gene prediction pipeline are displayed in the GUI that then provides additional information to allow for an intelligent, human-directed final assignment of ORFs as “authentic” genes. In the initial phase, ORFs with significant Pfam hits are scored as highly likely to be potential genes. Shorter ORFs entirely overlapping a longer ORF with a strong Pfam hit are scored much lower, as they are less likely to be true genes because of poxvirus gene structure features. Due to the comprehensiveness of Pfam, the majority of genes in any one species can be resolved in this manner. Evidence for other potential genes that did not receive high scores in the initial round can be visualized through the GUI, which when combined, allows a straightforward decision regarding gene assignment to be made. An ORF conserved in several different phylogenetically related species and having a predicted promoter motif present upstream of its coding region is much more likely to be a functional gene than an ORF lacking orthologs in other viruses, or lacking a promoter sequence. For a potential gene that is unique to a species, both the Glimmer score and the existence of a promoter-like motif provide strong clues to assist in determining whether it is an “authentic” gene. In addition, the precise translational start site can be determined based on the position of the predicted transcription start site, and comparison with orthologous genes in other strains and species.

## 4. Conclusions

Through the development and use of a new set of bioinformatics tools, we have re-annotated the gene sets of representative strains of all species in the *Poxviridae* genus *Orthopoxvirus*. These tools, packaged as the Poxvirus Genome Annotation System (PGAS) provide a semi-automated pipeline for the assessment of the coding potential of every ORF present in each poxvirus genome, and then present this information using a visualization tool that supports the final step of human-directed manual annotation of virus genes. An analysis of the coding potential for each gene of each genome emphasizes the variability of gene content in the orthopoxviruses. This variability is most apparent in genes that are functionally involved in various virus-host interactions and are located near the ends of the virus genome, while the central core region of the genome encodes genes that are much more conserved and are involved in basic virus replicative processes. Poxvirus evolution is mechanistically driven by single base changes and small insertions/deletions; acquisition of new genetic material through horizontal gene transfer; and changes in expression due to alterations in regulatory, promoter sequences. The importance of each mechanism in the evolution of any particular species probably reflects the various selection pressures that impact virus variation, biology, and response to the environment. This current work emphasizes the importance of gene loss in the overall divergence of orthopoxvirus species, and suggests that the loss of gene function through the deletion of genome sequences that no longer provide any selective advantage to virus replication is a major driving force supporting the variation and evolution of these viruses.

## Supplementary Materials



## Figures and Tables

**Table 1.** Orthopoxvirus genomes utilized for these analyses.

**Species**	**Strain Name**	**Abbreviation**	**Genome Length**	**Length of ITR**	**Haploid Gene Count**	**Genes in ITR**	**Genome GC%**	**Accession #**	**PubMed ID (Reference)**
*Camelpox virus*	Camelpox virus strain M-96 from Kazakhstan	CMLV	205,719	7736	188	3	33.2	AF438165 (NC_003391)	12033760 [73]
*Cowpox virus*	Cowpox virus strain Brighton Red	CPXV-BR	224,499	9710	209	5	33.4	AF482758 (NC_003663)	6961398 [74]
*Cowpox virus*	Cowpox virus strain Germany 91-3	CPXV-Ger	228,250	7374	211	5	33.5	DQ437593	16873609 [23]
*Cowpox virus*	Cowpox virus strain GRI-90	CPXV-Gri	223,666	8303	212	5	33.7	X94355	9568042 [75]
*Ectromelia virus*	Ectromelia virus strain Moscow	ECTV	209,771	9413	193	5	33.2	AF012825 (NC_004105)	14675635 [76]
*Monkeypox virus*	Monkeypox virus strain MPXV-WRAIR7-61; Walter Reed 267	MPXV-WR	199,195	8749	182	6	33.1	AY603973	16023693 [28]
*Monkeypox virus*	Monkeypox virus strain Zaire-96-I-16	MPXV-ZAI	196,858	6378	183	4	33.1	AF380138 (NC_003310)	11734207 [77]
*Taterapox virus*	Taterapox virus strain Dahomey 1968	TATV	198,050	4779	189	3	33.3	DQ437594 (NC_008291)	16873609 [23]
*Vaccinia virus*	Horsepox virus strain MNR-76	HSPV	212,633	7527	203	5	33.1	DQ792504	16940536 [78]
*Vaccinia virus*	Rabbitpox virus	RPXV	197,731	10022	192	6	33.5	AY484669	16227218 [79]
*Vaccinia virus*	Vaccinia virus strain Ankara	VACV-MVA	177,923	9644	174	2	33.1	U94848	9601507 [80]
*Vaccinia virus*	Vaccinia virus strain Copenhagen	VACV-Cop	191,738	11967	187	6	33.4	M35027	2219722 [58]
*Vaccinia virus*	Vaccinia virus strain WR (Western Reserve)	VACV-WR	194,711	10186	190	6	33.3	AY243312 (NC_006998)	
*Variola virus*	Variola virus strain Brazil 1966 (v66-39 Sao Paulo)	VARV-BRZ	188,062	518	180	0	32.7	DQ441419	16873609 [23]
*Variola virus*	Variola virus strain Kuwait 1967 (K1629)	VARV-KUW	185,853	522	179	0	32.7	DQ441433	16873609 [23]
*Variola virus*	Variola virus strain Sierra Leone 1969 (V68-258)	VARV-SLN	187,014	196	179	0	32.7	DQ441437	16873609 [23]
*Variola virus*	Variola virus strain South Africa 1965 (103 T’vaal, Nelspruit)	VARV-SAF	185,881	526	179	0	32.7	DQ441436	16873609 [23]

**Table 2.** Gene Content. The number of annotated genes with the indicated status.

**Genome**	**Gene Status**			
	Intact	Truncated	Fragmented	Missing
CPXV-GRI	209	3	0	2
CPXV-Ger	208	3	3	0
CPXV-BR	206	3	1	4
HSPV	181	22	4	7
ECTV	172	21	7	14
RPXV	179	13	4	18
VACV-WR	178	12	3	21
TATV	163	26	7	18
CMLV	174	14	8	18
VACV-Cop	172	15	4	23
MPXV-ZAI	176	7	14	17
MPXV-WR	175	7	14	18
VARV-BRZ	162	18	12	22
VARV-SLN	162	17	11	24
VARV-KUW	162	17	11	24
VARV-SAF	162	17	11	24
VACV-MVA	157	17	7	33
