# Supplementary material for: Orthopoxvirus Genome Evolution: The Role of Gene Loss
Source: Viruses. 2010 Sep 15;2(9):1933–67. doi: 10.3390/v2091933 (PMC3185746; doi:10.3390/v2091933)
Supplement: Supplementary file 2 [file viruses-02-01933-s002.pdf]

Supplementary Table 1. Comparative orthopoxvirus gene conservation.

This table provides detailed information on our re-annotation of 17 representative orthopoxvirus isolates. There is one row for each orthologous (syntelog) gene family, and several columns for each genome detailing the status, diploidy, strand, and location of ORFs in that syntelog group for that genome.

The rows of the table (syntelog families) are ordered by the genomic location of the CPXV-GRI homolog. In the two cases where there is no CPXV-GRI homolog, the family is placed in the correct syntenic position relative to its neighbors.

The first and last five syntelog families are given special treatment. These genes are located in the genomic ITRs of the CPXV isolates, and thus are diploid. We repeat these families at the beginning and end of the table, making 219 rows, though there are only 214 distinct haploid syntelog families (the diploid syntelogs are 1393 = 222274, 2286 = 221381, 3420 = 220247, 5392 = 218275, and 7652 = 216015.

Column Descriptions:

A	CPXV-GRI/GER Stop	Location of last nucleotide of the stop codon of the CPXV-GRI member of the syntelog family.<br><br>(*) In the two families lacking a CPXV-GRI homolog, the position of the CPXV-GER91 homolog is given.
B	POX tree	Y – Used for the phylogenetic analysis presented in figure 1.
C	OPV tree	Y – Used for the phylogenetic analysis presented in figure 6.
D	VACV-COP homolog	List of all VACV-COP (M35027.1) CDS, given via HindIII map names, that are in this syntelog family. In the case of diploid CDS (those present in the genomic ITR regions), the CDS for both copies are listed.
E	Expression Temporality	Expression temporality of the VACV-WR members of the syntelog group, based on microarray gene expression analysis [81], and our own ICM based prediction of early and late transcriptional promoter sites.<br>E – Early<br>I – Intermediate<br>L – Late<br>EL – Both early and late transcription.<br>Unk – no published evidence, and no prediction possible
E	Expression Evidence	lit – Expression temporality based on published experimental evidence<br><br>pred - Expression temporality based on our putative location of early and late transcriptional promoter sites.

Supplementary Table 1. *Cont.*

Column Descriptions:

	ISOLATE:status	Gene – gene is present and intact, and is at least 80% of the length of the longest CPXV homolog.<br><br>Trnc – gene is present, and the 5' end is conserved, but is less than 80% of the length CPXV sequence is present. Additional down-stream fragments my be present, also.<br><br>Frag – only fragments (pseudogenes) are present. The 5' end of the CPXV homolog is missing, and it is unlikely the remaining homologs are translated.<br><br>Miss – no homologous ORFs are present in this genome.
	ISOLATE:diploid	Dip – the gene is located in the genomic ITR region, and thus two identical copies of this gene are present, one at each end of the genome, and on opposite strand.<br><br>Hap – the gene is not located in the genomic ITR region, and thus has only one copy, though that copy may be broken into several adjacent fragments.
	ISOLATE:strand<br>ISOLATE:lend<br>ISOLATE:rend<br>ISOLATE:orf_start	The location of the syntelog group member in this genome, given in terms of its position on the positive strand of the genome.<br><br>When the gene status is "frag", then the location of the entire set of fragments is given.<br><br>If a syntelog group is diploid in this genome, but was not diploid in CPXV, then two lines of location will be given, one for the copy in the 5'ITR and one for the copy in 3'ITR.<br><br>The orf_start column contains the locaiton of the first available ATG codon when the translation start site has been changed from that codon to an internal codon based on promoter location and homology with other orthologs.
